# Overt and Covert Object Features Mediate Timing of Patterned Brain Activity during Motor Planning

**DOI:** 10.1093/texcom/tgaa080

**Published:** 2020-10-30

**Authors:** Michelle Marneweck, Scott T Grafton

**Affiliations:** 1 Department of Human Physiology, University of Oregon, Eugene, OR 97403-1249, USA; 2 Monash Biomedical Imaging, Monash University, Melbourne, Victoria 3168, Australia; 3 Turner Institute for Brain and Mental Health, Monash University, Melbourne, Victoria 3168, Australia; 4 Department of Psychological & Brain Sciences, University of California Santa Barbara, Santa Barbara, CA 93106, USA

**Keywords:** motor planning, neural representations, object manipulation, representational similarity analyses, ventral–dorsal

## Abstract

Humans are seamless in their ability to efficiently and reliably generate fingertip forces to gracefully interact with objects. Such interactions rarely end in awkward outcomes like spilling, crushing, or tilting given advanced motor planning. Here we combine multiband imaging with deconvolution- and Bayesian pattern component modeling of functional magnetic resonance imaging data and in-scanner kinematics, revealing compelling evidence that the human brain differentially represents preparatory information for skillful object interactions depending on the saliency of visual cues. Earlier patterned activity was particularly evident in ventral visual processing stream-, but also selectively in dorsal visual processing stream and cerebellum in conditions of heightened uncertainty when an object’s superficial shape was incompatible rather than compatible with a key underlying object feature.

## Introduction

The skillful handling of an object critically depends on perceptual input. Such input provides knowledge of multiple object properties including size, mass distribution, surface texture, and density. Knowing these features in advance allows for anticipatory force control and hence dexterous and efficient manipulation, including precise generation of lift forces. Knowledge of an object’s properties prior to manipulation can either be inferred directly from salient visual cues that are compatible with a relevant mechanical property (e.g., an asymmetric-shaped object with an asymmetric center of mass [CoM]) or it can be inferred indirectly from previous stored experiences (i.e., sensorimotor memories) when such congruency is not afforded (e.g., a symmetric-shaped object with an asymmetric CoM) (Schneider and Hermsdörfer 2016). Here, we localize brain areas that represent anticipatory planning of skilled hand-object interactions, and test whether these representations depend on overt object properties that can be directly inferred from salient visual cues or from covert features that are stored as sensorimotor memories.

Thirty years ago Goodale and Milner (1992) proposed the 2-stream model of cortical visual processing, bifurcating vision into a ventral stream (to occipitotemporal cortex) that uses vision for perception and a dorsal stream (to occipitoparietal cortex) that uses vision for action. Although such functional specialization and segregation exists to some degree when double dissociations between patients with brain damage are considered, it is increasingly apparent that in the healthy brain ventral–dorsal interactions (Takemura et al. 2016; Borra et al. 2017; Budisavljevic et al. 2018) and more widespread recruitment beyond both streams (e.g., cerebellum) are necessary for skilled object interactions (Cloutman 2013; van Polanen and Davare 2015; Milner 2017).

A growing number of reports show lateral occipital cortex (LOC) involvement during skilled object interactions particularly when overt visual information predicted from an object’s visual shape is irrelevant and covert object properties such as an asymmetric CoM must be estimated from previous experience (Gallivan et al. 2014; Marneweck et al. 2018; Klein et al. 2019; Marneweck and Grafton 2020a, 2020b). Thus, the ventral stream is involved in a hand-object interaction alongside frontoparietal regions in dorsolateral and dorsomedial streams that are more classically defined as subserving the reach and the grasp of a hand-object interaction, at least when objects have covert object properties. Involvement of ventral stream regions might be precursory for resolving the incongruency between overt (e.g., shape) and covert (e.g., CoM) object properties. On the other hand, such earlier input, either from ventral regions solely or in combination with dorsal and other regions, might be less necessary when visual cues of object properties are salient, given that dorsal stream can independently encode visual cue information of object properties (Fabbri et al. 2016; Bracci et al. 2017; Freud et al. 2017a, 2017b).

Here we examine evidence for the hypothesis of earlier ventral input (and beyond) when covert object properties are incompatible with that afforded by visual shape alone, compared with when object properties are overt and can be visually inferred online (Gallivan and Goodale 2018; de la Malla et al. 2019). Specifically, we identify whether the timing of activity patterns in brain areas associated with the planning of object manipulation depend on the availability and saliency of visual cues provided by the object. We obtained functional imaging data from 32 subjects while they grasped, lifted, and minimized the tilt of objects with an asymmetric (left- and right-sided) and symmetric CoM. Minimizing the tilt of an object with an asymmetric CoM requires precise anticipatory force control, by generating a torque in the opposite direction of the object’s off-centered torque, with an asymmetrical, differential partitioning of thumb and index finger lift forces at lift onset. We manipulated the extent to which visual cues were congruent with the CoM by using 2 differently shaped objects: L-shaped and T-shaped. For an L-shaped object with a hidden mass positioned at the tip of the horizontal segment the visual cue is congruent with the predicted CoM based on the object shape. This can be contrasted with a hidden mass positioned at the base of the vertical segment which is less congruent with the expected CoM based on shape alone. For a T-shaped object, a hidden mass positioned at the base of the vertical segment is congruent with the predicted mass distribution whereas a hidden mass at the tip of either horizontal segment is incongruent with the predicted CoM based on the object shape. Using multiband functional magnetic resonance imaging (fMRI), in conjunction with object kinematics and a deconvolution-based general linear modeling (GLM) approach, we used these different conditions to track multivoxel patterns of cortical and subcortical activity during anticipatory planning for manipulating objects with overt and covert CoMs defined by its congruency with the shape of the object. Bayesian variational representational similarity analyses (vRSA) then identified condition-specific contrasts of multivoxel patterned activity in PMv, AIP, SPL7, and PSC (hereafter referred to as dorsal regions) and LOC and pFG (referred to as ventral regions), and cerebellum.

Previously we established that there are differences of multivoxel patterns of activity in the above brain regions that emerge just prior to the successful lifting of T-shaped objects when we contrasted pattern distances for asymmetric left and right CoMs (Marneweck et al. 2018; Marneweck and Grafton 2020a, 2020b). Critically, the object’s visual shape did not allude to the covert CoM. These patterns became more different or distinct from each other (i.e., CoM specific) as behavioral performance improved (Marneweck and Grafton 2020*a*), suggesting regions with CoM-specific patterns rapidly learn to represent covert CoM information that is relevant for guiding anticipatory force control (which vary for objects with left and right CoMs) (Table 1; Covert CoM contrast of manipulating a T-shaped object with left and right CoM). Here, we then consider how multivoxel spatial patterns differ for conditions where the object incorporates covert or overt CoM properties (Table 1; Overt CoM contrast of manipulating a L-shaped object with left and right CoM). Specifically, we test a hypothesis that CoM-pattern differences are magnified in early time bins for anticipatory control of objects that have covert CoMs (a hidden, asymmetric CoM), and in later time bins for anticipatory control of objects with overt CoMs.

**Table 1:** Contrasts examining the evidence that multivoxel patterned activity varies between conditions with overt and covert CoMs, visual shape and visual shape-CoM congruency differences, respectively, and with subtler magnitude-based differences in torque force distribution and its interaction with visual shape

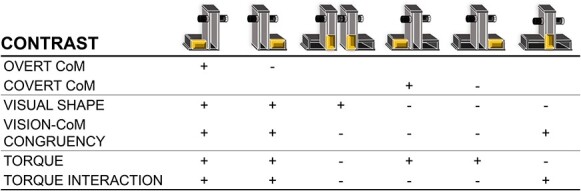

By testing 2 sets of object shapes with either congruent or incongruent CoM locations the pattern component modeling framework provides us with the opportunity to consider several alternative models that could explain multivoxel pattern distances at any given time period (Table 1). First, we could test for the interaction of the visual congruence between object shape and expected CoM (Table 1; Vision—CoM Congruency; contrasting conditions with congruent and incongruent shape and CoM information, irrespective of shape or the existence/direction of torque). In this case, the comparison is irrespective of the shape of the objects or the existence/direction of torque, since conditions in both variables have L- and T-shaped objects with centered and off-centered weights. The contrast reflects the participant’s appraisal that the shape of the object (irrespective of the particular shape) is consistent with the expected CoM of the object or not. If this contrast dominates in pattern component modeling in early time points in ventral regions, for example, then it suggests that under situations where the superficial shape of the object is incompatible with the actual CoM (and subjects must primarily draw on prior sensorimotor memories with that object), then the ventral stream input contributes early in lift force planning. Second, we could determine if multivoxel pattern differences during early and late time periods were simply a result of visual shape (irrespective of CoM and congruency; Table 1; visual shape; contrasting L-and T-shaped objects). Finally, we could test if pattern differences between conditions were influenced by the amount of torque the 2 lifting digits would need to generate, irrespective of object shape or congruency (Table 1; torque; contrasting center- and off-centered weighted conditions, irrespective of shape or its congruency with the CoM), and whether such pattern differences were sensitive to object shape (Table 1; torque interaction; evaluating if a center- vs. off-centered patterned distance varies depending on the object’s shape).

## Materials and Methods

### Participants

Thirty-two right-handed healthy adults (median age: 21; range: 18–27; 17 females) with normal or corrected to normal vision participated in this study, and gave informed consent. The study was approved by the Human Subjects Committee, Office of Research, University of California, Santa Barbara, and all guidelines were followed.

### Materials

**Figure 1:**
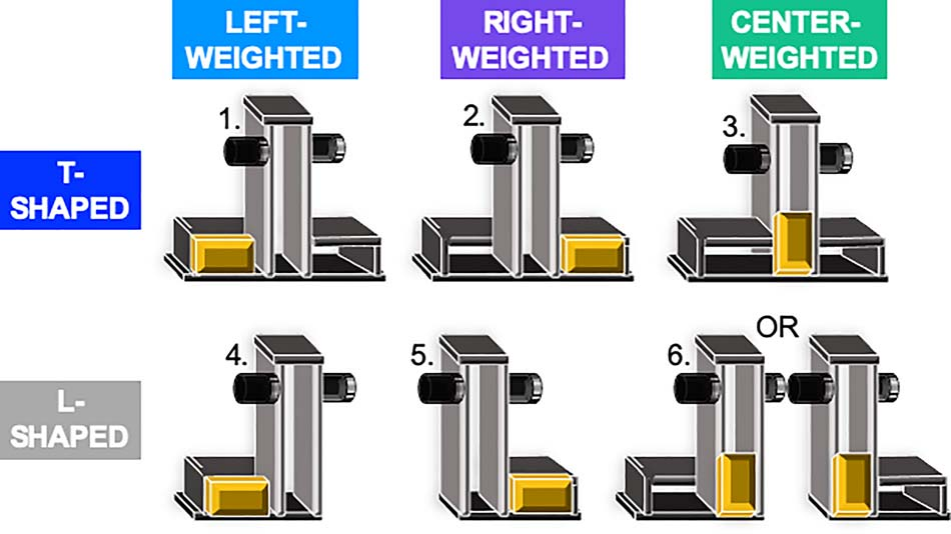
The objects and conditions. Schematic illustration of the custom-made objects and 6 experimental conditions in which subjects were to minimize roll while lifting a T-shaped (1) left, (2) right, and (3) center-weighted object and while lifting an L-shaped (4) left, (5) right, and (6) center-weighted object. The center-weighted L-shaped object faced either to the left or the right, and was counterbalanced between subjects.

We custom-made MRI-compatible L-shaped and inverted T-shaped Plexiglass objects (see Fig. 1). Each object had circular grasp surfaces (diameter: 1.5 cm; between grasp distance: 8.0 cm) attached on each side of a vertical column (height: 13.0 cm; width: 3.4 cm; depth: 5.0 cm) and a horizontal base. This base extended to both sides of the vertical column for the T-shaped object, and to one side only for the L-shaped object (each side: height: 0.5 cm; width: 7.3 cm; depth: 5.0 cm). A lead block (height: 2.7 cm; width: 5.0 cm; depth: 3 cm; mass: 441 g) was placed on the horizontal base on one side of the vertical column creating an off-centered mass distribution or in the center of the vertical column creating a centered mass distribution. The lead block was concealed by covers. The total mass of each object was 688 g. When the mass was off-centered, the torque was 223 Nmm.

A wooden table was placed over each participant’s hips such that the object and the button box on the table were at arm’s reach. The object was angled 30° in a counterclockwise direction with respect to the frontal plane, which pilot work confirmed would minimize the wrist’s biomechanical constraints that influence object roll in a supine position (e.g., wrist stiffening). With a mirror attached to the head coil, participants had full view of the object, button box, and their hands at all times.

Object roll and vertical height was measured with a 2-camera MRI-compatible motion tracking system (Precision Point Tracking System; Worldviz; frame rate: 150 Hz; camera resolution: 640 × 480 VGA; spatial accuracy at focal distance: submillimeter) and with 2 near infrared LED markers that were affixed to the vertical column of each object.

### Experimental Design and Procedure

The experiment had 6 within-subject conditions (see Fig. 1): manipulating a T-shaped (1) left, (2) right, and (3) center-weighted object and manipulating an L-shaped (4) left, (5) right, and (6) center-weighted object, with the aim of minimizing the object roll at all times. Standardized instructions and 10 practice trials were given, the latter of which involved lifting a water bottle to the task’s audio cues. On each trial, subjects pressed a button with the palm of their right hand in a relaxed position until an audio cue instructed them to reach, grasp and lift the object, hold it at the height of a marker (4 cm) until a second audio cue (4 s after the first audio cue) instructed them to place the object back in its original position (marked with adhesive tape) and return their hand to the button. A third audio “error” cue would subsequently sound if the object rolled more than 5° in either direction during the trial.

Participants lifted the T- and L-shaped objects for a total of 80 trials (40 trials each in 2 functional runs, each of which lasted ~12 min). Within each run, the 3 CoM conditions were blocked in trials of 4 (10 blocks total). After each block, the experimenter remained inside the magnet room for the duration of each run and shifted the CoM, and informed the participant of the new CoM in writing on a cardboard sign. Subjects were free to direct their gaze through the workspace throughout the trial, but the cardboard sign indicating the upcoming CoM between blocks prevented subjects from seeing the object being changed. An intertrial interval randomly chosen to be 2, 3, 4, 5, or 6 s, with a rest period between each of the within-run CoM blocks (during which time the experimenter changed the CoM). The duration of the rest periods was approximately 30 s (depending on the time it took the experimenter to change the CoM and provide the instruction for the upcoming block of trials). There were 16 trials for each of the off-center weight conditions, and 8 trials for the center weight condition. The rationale for varying the trial number for off-center and center-weighted conditions was to have a similar number of successful trials for each condition (with more errors for the off-center weighted conditions expected). Subjects made 0 to 7 errors (median = 1) in the off-centered conditions and 0 to 2 errors in the centered condition (median = 0). It is possible that a smaller number of trials in a given condition to give way to larger variability. However, this was less of a concern in this case where the center-weighted condition with a smaller number of trials were behaviorally consistent. Trials of the center-weighted condition were either done at the start or end of the run, and the order of the off-center weighted conditions, and the order of the L- and T-shaped conditions, were counterbalanced. The center-weighted L-shaped object faced either to the left or the right. Its direction was either congruent or incongruent with the condition that followed or preceded it, and this was also counterbalanced between subjects.

Structural and functional MRI data were collected using a Siemens 3 T Magnetom Prisma Fit (64-channel phased-array head coil). Following high-resolution 0.94 mm isotropic T1-MPRAGE (repetition time (TR) = 2500 ms; echo time (TE) = 2.2 ms; flip angle (FA) = 7°; field of view (FOV) = 241 mm) sagittal sequence images, subjects manipulated the L- or T-shaped object during which BOLD contrast was measured with a CMRR multiband (University of Minnesota) T2*-weighted echo planar gradient-echo imaging sequence (TR = 400 ms; TE = 35 ms; FA = 52°; FOV = 192 mm; multiband factor 8).

### Kinematic Data Processing and Analyses

Kinematic data were filtered with a fourth-order Butterworth filter with a cut-off frequency of 5 Hz. Object lift onset was defined as the time that the vertical position of the object exceeded 1 mm and remained above this value for 20 samples. Object roll was defined as the angle of the object in the oblique plane. Peak object roll was recorded shortly after lift onset (~250 ms) before feedback of the object properties could be used to counter the object roll. A trial was considered an error when object roll in this time window exceeded 5°. These trials were excluded from the analyses.

To rule out that multivoxel pattern differences are a result of behavioral performance differences between the CoM and object shape conditions, we ran a two-way ANOVA to examine the effect of CoM (left-, right-, and center-weighted) and object shape (L- and T-shaped objects) on object roll. Multiple comparisons were corrected using Bonferroni pairwise comparisons. For consistency between our behavioral and MRI analyses, we also conducted a Bayesian ANOVA to assess the evidence for the null model over alternative models (i.e., Shape effect; CoM effect; Shape + CoM effect; Shape + CoM + Shape*CoM) using the BayesFactor package in R.

### MRI Data Processing and Analyses

MRI data were preprocessed and analyzed using SPM12 (Wellcome Trust Center for Neuroimaging, London, UK) and FSL (https://fsl.fmrib.ox.ac.uk/fsl/fslwiki/)(Jenkinson et al. 2012). Using SPM, subjects’ functional images were spatially realigned to a mean image from each run using second-degree B-spline interpolation, and coregistered to the T1. ArtRepair v5b3 (art_global.m) was used to inspect and repair using linear interpolation from the nearest unrepaired scans any outlier volumes exceeding the intensity variation threshold (2 mm) or the intensity variation threshold (2.7% more than the mean global signal intensity) (Mazaika et al. 2005; Mazaika et al. 2007; Mazaika et al. 2009). Between-subject normalization for the cerebellum was done with the SUIT SPM toolbox (Diedrichsen 2006; Diedrichsen et al. 2009; Diedrichsen et al. 2011; Diedrichsen and Zotow 2015), and for the rest of the brain with SPM’s normalize function.

Multivoxel spatial patterns between conditions were compared using vRSA (Friston et al. 2019) with an adaptation of the DEMO_CVA_RSA.m script available in SPM12. This method assesses the extent to which a given condition or contrast between conditions might contribute to patterns of responses that are spatially distributed across a set of voxels in a given region of interest (ROI). First, we computed a deconvolution GLM in SPM for each run separately, with the RobustWLS Toolbox selected to downweight volumes with high noise variance to further account for movement artifact. Left, right-, and center-weighted conditions, and an error condition, were entered as predictor variables, the latter of which was not analyzed further. In selecting a finite impulse response (FIR) function, we modeled 9 × 800-ms time bins of activity for each of the conditions, with the onset of the first FIR bin set to 800 ms before lift onset (window length: 7.2 s; order: 800 ms). In this way, we sufficiently tracked activation before lift onset and through the peak of the hemodynamic response relating to lift onset, which was assumed to occur 4–6 s after lift onset. Thus, we investigated brain activity predominantly occurring during a prelift time window when anticipatory forces are initiated to generate an appropriate torque that minimizes object roll. The same procedure has been used in our previous studies (Marneweck et al. 2018; Marneweck and Grafton 2020a, 2020b).

**Figure 2:**
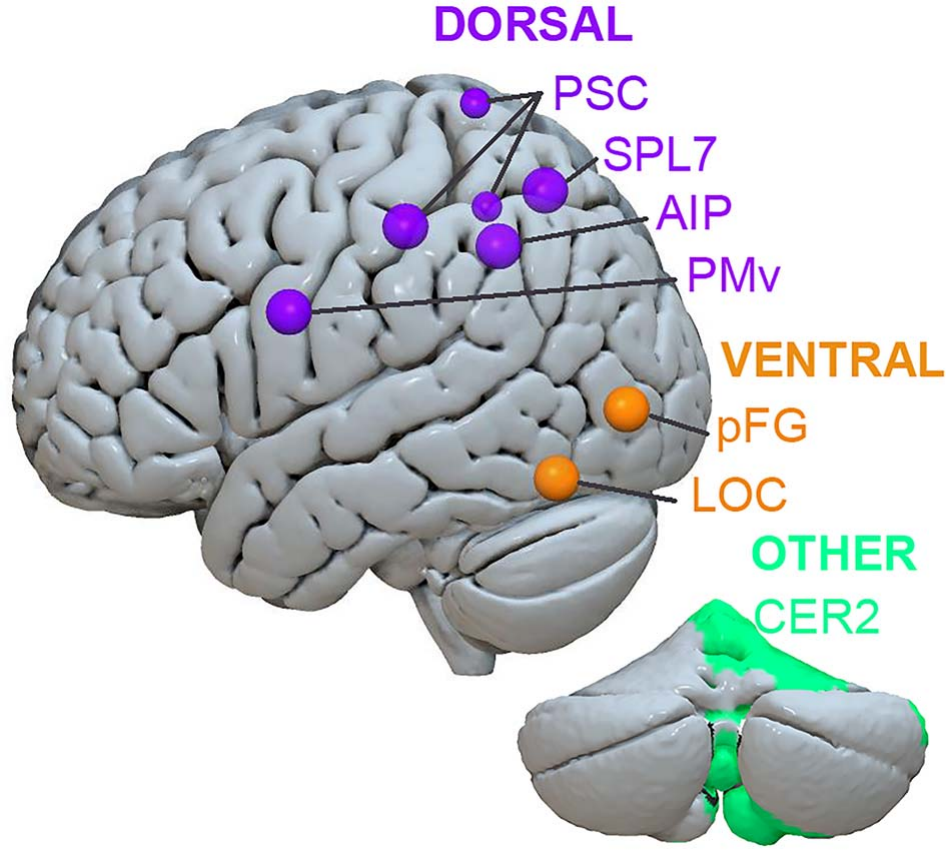
Regions of interest. Predefined ROIs displayed on the MNI-152 atlas using visualization software, Surf Ice (https://www.nitrc.org/projects/surfice/). Abbreviations: PMv, ventral premotor; PSC, primary somatosensory cortex; SPL7, superior parietal lobule 7; AIP, anterior intraparietal area; LOC, lateral occipital cortex; pFG, posterior fusiform gyrus; CER2, functional cerebellar region 2. All cortical regions (including those on the medial wall) are projected to the lateral surface.

GLM-derived β values for each of the conditions at each of the FIR time points were extracted from ROIs using FSL’s fslmeants. Predefined cerebellar and cortical ROIs that have previously been shown to be sensitive to differences when manipulating inverted T-shaped objects of different torques (Marneweck and Grafton 2020a, 2020b): SPL7, anterior intraparietal area (AIP), primary central sulcus (PSC/SI), and LOC were extracted from the SPM Anatomy Toolbox (Eickhoff et al. 2005; Eickhoff et al. 2006; Eickhoff et al. 2007). Ventral premotor area (PMv) were free-drawn on a standardized surface mesh in SUMA (Saad et al. 2004) based on predefined anatomical parcellations (Geyer et al. 1996; Picard and Strick 2001; Tomassini et al. 2007; Destrieux et al. 2010), which were projected to standard MNI space and mapped backed to the subject’s T1-weighted image (Barany et al. 2014). Cerebellar ROI 2 was extracted from a recently published cerebellar functional atlas (King et al. 2019). In addition, we extracted another ventral region, posterior fusiform gyrus (pFG), given its role in object recognition (pFG1, 2, and 4; from the SPM Anatomy Toolbox). See Figure 2 for ROIs mapped onto a cortical surface.

To summarize, for each condition and time bin, we now had a set of GLM-derived β values that make up a multivoxel spatial pattern in a given ROI, which we next import for model comparisons in vRSA. The vRSA approach starts like a more classical RSA approach in its comparison of between-condition differences in spatial voxel patterns in a given ROI. In vRSA, the relationship between condition- or stimuli-specific spatial voxel patterns are described in terms of second-order similarity or covariance matrices (whereas in classical RSA they are expressed in terms of a representational dissimilarity matrix). With these comparisons, vRSA can be used to assess the contribution of multiple contrasts of conditions to a given ROI’s response pattern (taking into account all proposed contrasts). In particular, it evaluates if the posterior probability of underlying response pattern differences is consistent with linear contrasts of the conditions. The outcome of each contrast is a log evidence value (i.e., Bayes factor), which quantifies the evidence for a given contrast to contribute to activity pattern differences in a given ROI. The Bayes factor is a fundamental part of the Bayesian approach to testing hypotheses, which, dissimilar to fixed significance levels of frequentists approaches, provides a continuous degree or measure of evidence for the null and alternative hypotheses, H0 and H1 (see Dienes Z and McIatchie N 2018, for a comparison between significance testing and Bayes factor). When the Bayes factor = 1, the evidence does not favor either H0 and H1 with both models predicting the data equally well. The H1 model is favored over H0 when the Bayes factor increases beyond 1 (toward infinity), and the H0 model is favored over H1 when the Bayes factor falls below 1 (toward zero). A Bayes factor of approximately 3 has been suggested to match a “substantial” amount of evidence that a contrast of interest contributes to a region’s observed response pattern (Kass and Raftery 1995; Jeffreys 1998). Most of our log evidence values exceeded 30, matching a “strong” amount of evidence for a contrast to contribute to region’s observed response pattern.

Table 1 summarizes the contrasts entered into vRSA model comparisons to assess the evidence for activity pattern differences in each ROI and time bin between left- and right-weighted conditions when visual shape cues were available and congruent with the CoM (i.e., L-shaped manipulation; overt CoM) or masked and incongruent with the CoM (i.e., T-shaped manipulation; covert CoM). Note, the hidden mass that defines the CoM was never visible; instead it was either made to be overt or covert by its congruency or incongruency with the visual shape of the object. We then combined log evidence values consistent with substantial to strong evidence for a contrast to contribute to a region’s response pattern into a frequency distribution plot for the overt and covert CoM contrasts, respectively. With cumulative Gaussian functions fitted to each plot, we compared the peak amplitude, the width, as well as the mean time point at which the peak amplitude occurred for each contrast. Since varying multivoxel pattern differences for overt and covert CoM contrasts could be driven by either visual shape cues or the extent of congruency between the shape cue and the CoM, we ran 2 additional contrasts. The first contrast compared multivoxel patterns between conditions with congruent and incongruent visual cues and CoM, while canceling out any shape or CoM effects (i.e., vision–CoM congruency contrast). The second contrast (visual shape) compared multivoxel patterns between conditions in which the shape varied, canceling out any effects of congruency or CoM. Finally, with the inclusion of a center-weighted condition in our study, we were able to evaluate for the first time whether these regions might be sensitive to subtler between-condition lift force differences (torque contrast). We also check whether any such subtler effects were dependent on the shape of the manipulated object (torque interaction contrast).

## Results

Thirty-two participants reached, grasped, and lifted while minimizing the tilt of an L-shaped and a T-shaped object with the CoM on the left, right or in the center during fMRI acquisition (Fig. 1). Cortical and subcortical activity for each condition was estimated using first-level deconvolution-based GLM in 800-ms time bins for 7.2 s starting 800 ms before lift onset, which, given the 4–6 s hemodynamic response delay, predominantly tracked prelift onset activity as per previous work (Marneweck et al. 2018; Marneweck and Grafton 2020a, 2020b). Table 1 shows the 3 sets of contrasts of condition-specific activity that were entered into vRSA within each time bin and ROI. This method is used to evaluate the evidence that a given between-condition contrast contributes to distinct multivoxel patterned activity as was done previously (Marneweck and Grafton 2020a, 2020b).

First, the overt CoM and covert CoM contrasts examined whether multivoxel pattern distances vary between L- and T-shaped object manipulation in which the CoM is either (1) overt and congruent or (2) covert and incongruent with the object’s visual shape. Second, the visual shape and visual–CoM congruency contrasts examined whether multivoxel patterns varied between conditions that vary in visual shape (irrespective of CoM and congruency) and between conditions with incongruence and congruence in visual shape and CoM (irrespective of CoM and visual shape). Finally, the torque contrast checked for multivoxel pattern differences between conditions with subtler differences in lift force distribution between manipulating objects with CoMs that were off-centered (requiring torque) and centered (requiring no torque), and whether any such effects depended on the shape of the object (torque interaction).

### Behavioral Performance

**Figure 3:**
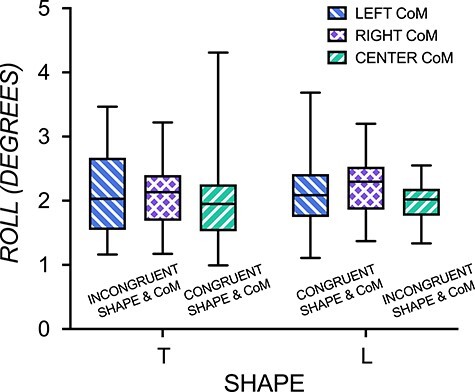
Behavioral results. Similar performance when lifting T- or L-shaped objects with their center of mass (CoM) on the left (blue), right (purple), and in the center (green). Box and whisker plot bars depict minimum to maximum values.

As Figure 3 shows, behavioral performance across the 6 conditions shown in Table 1 were similar (with no effects of object shape, interaction, and a significant yet small effect of CoM (*F*(2,66) = 3.62, *P* = 0.03 ηp^2^ = 0.01) that did not survive the Bonferroni correction). A Bayesian ANOVA was consistent with the classical ANOVA analysis, with substantial to strong evidence favoring the null model over the alternative models (Shape effect: BF_01_ = 5.22; Shape + CoM effects, BF_01_ = 3.68; Shape + CoM + Shape*CoM: BF_01_ = 30.81) and weak, anecdotal evidence for a CoM effect (BF_10_ = 1.39). To rule out a possible congruency effect, we found no effect of congruency or interaction, and a small effect of CoM (*F*(2,66) = 3.62, *P* = 0.03, ηp^2^ = 0.01) that did not survive a Bonferroni correction. Therefore, the results from the contrasts described below are not confounded by behavioral performance differences.

### Timing-Based Patterned Activity Differences When Planning to Manipulate Objects with Overt and Covert CoM Properties

**Figure 4:**
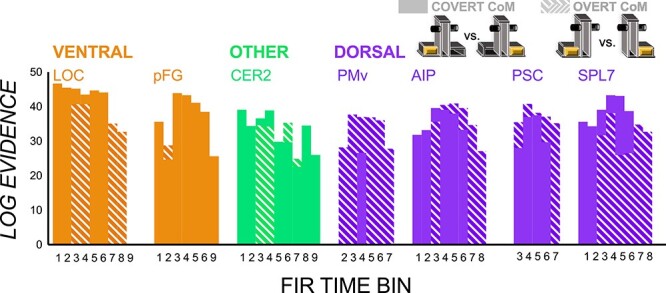
vRSA results from the overt and covert contrasts. Log evidence values indicating substantial to strong evidence for multivoxel pattern differences between planning of manipulation of objects with left- and right-CoMs when they are covert (solid) and overt (dashed).

Figure 4 shows vRSA-computed log evidence (Bayes factor) values indicating substantial (>3) to strong (>30) evidence for between-CoM multivoxel pattern differences when the off-centered CoM is overt and covert. It is clear that all regions at some point were sensitive to both overt and covert CoM contrasts. However, the timing of this sensitivity varied. Between-CoM-pattern differences were generally magnified in early time bins in the covert CoM contrast and in later time bins in the overt contrast. Early covert CoM-specificity in patterned activity was most prominent in ventral regions, pFG and LOC, but also in early time bins for cerebellum, AIP and SPL7.

**Figure 5:**
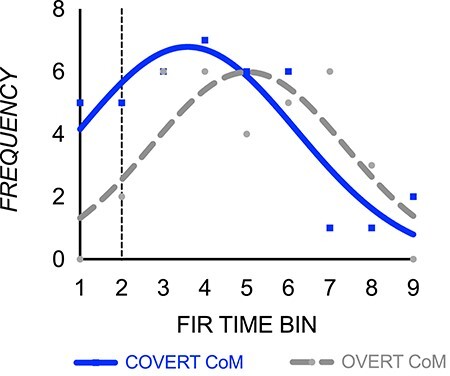
Across-ROI frequency of overt and covert CoM-specific patterned activity. Cumulative Gaussian functions fitted to a frequency distribution plot of log evidence values > 3, indicating substantial evidence for a contrast of covert (solid blue) and overt (dashed gray) left- and right-weighted conditions contributing to multivoxel pattern differences. The *y*-axis reflects the number of regions with contrasts showing substantial evidence for patterned activity differences between left- and right-weighted conditions. Lift onset is depicted by a black vertical dotted line.

To characterize this temporal difference for overt and covert CoMs further we aggregated Bayes evidence across all regions for the overt and covert CoMs. Figure 5 highlights this temporal shift in the bulk of CoM-pattern sensitivity at differing time points for manipulating objects with covert and overt CoMs. Cumulative Gaussian functions fitted to the frequency distribution data (overt CoM *r*^2^ = 0.68; covert CoM *r*^2^ = 0.78) showed that the peak amplitude (i.e., the number of regions with differential responses for left- and right-weighted conditions) was similar (overt = 5.98, 95% CI = 3.76–8.39; covert = 6.79, 95% CI = 5.00–8.76). The standard deviation of the distribution was also similar (overt = 2.32, 95% CI = 1.56–4.40; covert = 2.61, 95% CI = 1.78–5.34). However, the mean time of peak amplitude was earlier for the manipulation with covert than overt properties (i.e., time when the bulk of regions showed differential response patterns; overt = 5.03, 95% CI = 3.83–6.30; covert = 3.59, 95% CI = 1.04–46). Together these results confirm earlier magnified patterned activity in ventral, cerebellar, and select dorsal regions when planning to manipulate objects with covert CoMs, and later magnified patterned activity (predominantly in dorsal regions) for that with overt CoMs.

### Distinct Patterned Activity Depends on Congruence between Shape and CoM

**Figure 6:**
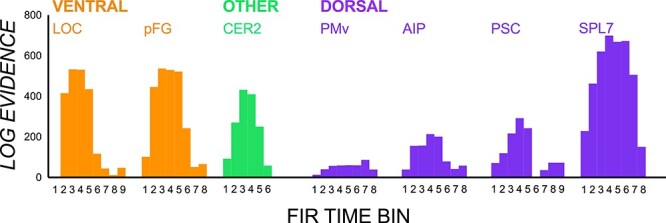
vRSA results from the vision-shape congruency contrast. Log evidence values in each ROI and time bin giving evidence for contrasts of conditions with congruence and incongruence between visual shape cues and center of mass to contribute to multivoxel pattern differences.

Earlier patterned activity for manipulating objects with covert CoMs suggest that precursory input from ventral stream areas (and beyond) is necessary when object properties are incompatible with that afforded by visual shape. To strengthen this conclusion, a vision–CoM congruency contrast (see Table 1) was run to examine pattern differences between conditions with incongruence and congruence in visual shape and CoM (irrespective of independent effects of CoM and visual shape). Results are displayed in Figure 6. All ROIs were sensitive to these congruency differences at some point, but this effect was particularly prominent during early time bins in ventral stream regions followed by cerebellar regions and SPL7. Interestingly, these pronounced pattern differences are in the same regions that showed magnified pattern differences during planning of manipulating objects with covert CoMs. These findings fit with the idea that these regions are involved in the early encoding of processes relating to vision–CoM incompatibility and subsequent memory-based estimation of object properties.

### Distinct Patterned Activity for Visual Shape

**Figure 7:**
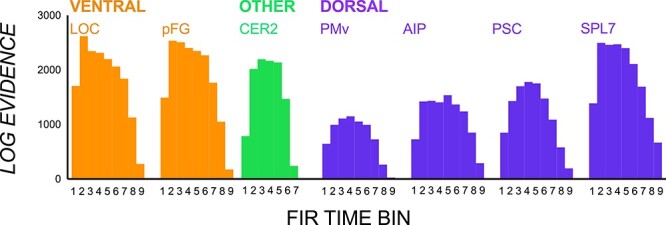
vRSA results from the visual shape contrast. Log evidence values in each ROI and time bin giving evidence for contrasts between conditions with and without visual shape differences to contribute to differing multivoxel patterns.

To support the claim that manipulating objects with salient visual cues may rely less on these earlier inputs from ventral stream and beyond, we ran the visual shape contrast, evaluating whether patterns varied between conditions with and without visual shape differences (irrespective of CoM and congruency; see Fig. 7). The critical finding here was both ventral and dorsal regions show sensitivity to visual shape cue differences, even when the CoM and congruency effects are accounted for.

Altogether, these results suggest that manipulation of objects with incongruence or incompatibility of object properties might be processed by earlier input by ventral regions, select dorsal regions and cerebellum, whereas manipulation of objects with salient visual shape cues can be encoded within dorsal stream.

### Distinguishable Patterns for Objects with and without Torque

**Figure 8:**
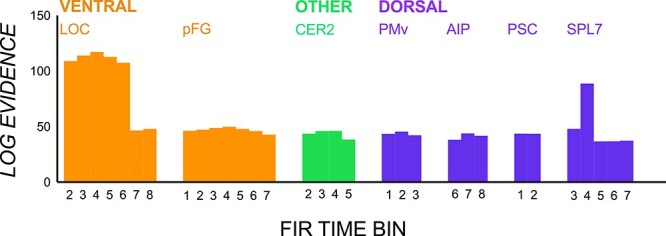
vRSA results from the torque contrast. Log evidence values giving substantial to strong evidence that manipulating off-center and center-weighted objects contribute to different multivoxel patterns.

In this and previous studies we investigated CoM-specific patterns by comparing conditions in which subjects were required to differentially partition digit forces to generate compensatory torques in opposite directions. Behavioral success (i.e., roll minimization) on the 2 contrasting conditions requires vastly different index finger and thumb lift forces (e.g., in the left CoM condition, subjects generate more force by the thumb than index finger whereas in the right CoM condition, subjects generate more force by the index finger than the thumb). This raises a question as to whether pattern differences are representing the relative degree of direction-specific torque controlled by both digits. As shown in Figure 8, results from the torque contrast show that ROIs also showed different pattern signatures when preparing to lift an object with and without torque, the former requiring differential and the latter uniformly distributed forces. Results from the torque interaction contrast were predominantly null, suggesting that these effects did not interact with object shape. Overall, these results confirm that ROIs are not only sensitive to planning a directional-specific torque but also the torque magnitude and/or individual digit lift force.

## Discussion

Here, we used Bayesian vRSA of deconvolution-modeled fMRI data to compare patterned activity in the brain when planning to skillfully lift objects with off-centered mass distributions, with and without the availability of congruent visual shape cues that alluded to the CoM location. First, we replicate our previous work (Marneweck et al. 2018; Marneweck and Grafton 2020a, 2020b) showing distinct patterned activity when contrasting planning of objects with covert off-centered weights that were incongruent to its visual shape (left vs. right CoM with a T-shaped object). Second, we extend this result by showing distinct patterned activity when planning to lift objects with overt off-centered weights that were congruent to its visual shape (left vs. right CoM with an L-shaped object). Third, we further show distinct patterned activity when contrasting objects with centered- and off-centered weights. The latter 2 results extend our previous work by showing that these regions not only plan a directional-specific torque of objects with covert properties, but they also plan lifts for objects with overt properties. Furthermore, the magnitude of the torque and/or the individual digit lift force are also represented in these areas. Most significantly, and consistent with our hypotheses, planning the lift of objects with off-centered weights in the absence of salient congruent visual cues contributed to an earlier emergence of CoM-specific pattern distances, most prominently in ventral visual stream regions as well as in cerebellar and select dorsal stream regions. Early patterned activity differences were also most notably seen in ventral stream regions when contrasting conditions with and without shape-CoM congruency irrespective of CoM and shape differences. Altogether the findings suggest that there are nuances in the way that the brain encodes anticipatory control of lift force, depending on the availability of salient visual shape cues. Early ventral stream input seems necessary for lift force planning in more uncertain situations where an object’s superficial shape is incompatible with its actual CoM, thereby requiring increased reliance on prior sensorimotor memories with that object.

The main question we sought to answer in this study was whether the brain differentially processes anticipatory planning of skilled object manipulation depending on the availability and saliency of visual shape cues. In some respects, our results suggest that there is little difference in how the brain encodes anticipatory control with and without salient visual cues. The same regions as that shown previously (Marneweck and Grafton 2020a; 2020b) showed CoM-specific patterns in both cases and the duration of these distinctive patterns across ROIs were also statistically indistinguishable. There was also more or less the same amount of model evidence for CoM-specific patterns in both cases in middle time bins (e.g., bins 3–4 in LOC, bin 2 in pFG, bins 3–4, 6–7 in cerebellum, and bins 3–6 in AIP, PSC, and SPL7; see Fig. 4). This suggests that CoM-pattern distances in both cases reflect successful planning of object lifts similarly, particularly in time bins closer to lift onset (which is conceivably closer to the point at which the torque and force generation plan would be in place). On the other hand, there were differences in the timing of when these CoM-specific patterns emerge, which depended on the availability and saliency of visual shape cues.

The timing result, showing an earlier emergence of CoM-specific patterns for T-shaped object manipulation planning, suggests the need for more and earlier input by mostly ventral regions in the absence of a visual shape cue. That the same regions with earlier CoM-specific patterns are also sensitive to shape-CoM congruency differences strengthens the supposition that this earlier ventral input contributes to reconciling the CoM-shape incongruence, suppressing the use of the visual shape cue, and therein relying predominantly on a sensorimotor memory-based anticipatory plan to achieve the task goal. Ventral stream involvement for recalling sensorimotor memory-based information is also supported by TMS over LOC resulting in grasp kinematic modulation during a delayed grasp onset paradigm (Cohen et al. 2009).

Consistent with the idea of temporal-based differences between planning of object manipulation with and without salient visual cues, a TMS study showed that when visual information is unavailable, corticospinal excitability is scaled according to the weight of the object from the previous lift as early as 50 ms after object presentation (and at 100 ms and 150 ms). In contrast, when visual information is available, this effect remains present at 50 ms but becomes gradually suppressed at 100 ms and is completely abolished 150 ms later (Loh et al. 2010). Like we show here, these findings suggest that the motor system is recruited at differential timescales when visual cues are absent or incongruent with a key object feature than situations where such cues can be used to guide skilled action.

Importantly, we showed that these timing-based differences are not a result of behavioral performance differences, since these outcome measures were matched in our conditions. However, it is important to note that we modeled trials after subjects had acquired familiarity with objects and object manipulation was successful. The same matched behavioral success is not consistent during early sensorimotor learning. For example, subjects perform poorer in their early attempts to minimize role of a T- than L-shaped object with an incongruent than congruent CoM (Gordon et al. 1993; Salimi et al. 2003; Lee-Miller et al. 2016). Similar results of incongruence adversely affecting perception of weight and force control is seen in early exposure to size-weight illusions (Saccone and Chouinard 2019). Previously, we have showed that sensorimotor learning was accompanied by an increase in CoM-pattern specificity when manipulating objects with shape-CoM incongruencies, particularly in early time bins (Marneweck and Grafton 2020a). Specifically, we show that the CoM-specific distances increase with repeated CoM exposures (i.e., when comparing the last trial of a given CoM and the first trial after the CoM switch). This distance increase with repeated CoM exposures is associated with improved behavioral performance on the first trial after the successive CoM switch (and these increased pattern distances remain present 24 h later, in line with behavioral consolidation effects). Our previous study (Marneweck and Grafton 2020a) and behavioral studies of others (e.g., Fu et al. 2010; Zhang et al. 2010) show this initial training and retraining (after a switch) is exceptionally swift and that CoM-specific patterns emerge on a very rapid time scale (within a given block of trials). We do not see within-COM changes in patterned activity over longer time scales (within a block (Marneweck and Grafton 2020a) or between runs (Marneweck et al. 2018)). That is, the representation for a given CoM that one has become familiar with is stable. The current study findings suggest the ventral stream can generate a detailed representation about the hidden attributes of an object after a very short number of exposures to that object. It might be that such earlier ventral inputs are precursory to achieving the same level of task success and stability as that which can be achieved with less such inputs when visual cues allude to the CoM. Since we excluded errors and we are modeling activity at the onset of lift, our contrasts of successful trials are more likely reflecting the representation of newly acquired sensorimotor memory information than error correction or learning.

It is becoming increasingly recognized that when interacting with objects, the brain operates probabilistically using Bayesian inference in optimizing its perception of objects, many of which are signaled and must be integrated efficiently by more than one sense (or memory) that sometimes would offer incongruent accounts (Körding and Wolpert 2004; Yang et al. 2012; Darlington et al. 2018; Alais and Burr 2019). The central nervous system is thought to integrate these cues by a weighted linear sum where each cue is inversely weighted based on its variance or uncertainty, therein producing an integrated sensory estimate with minimal uncertainty and maximized perceptual precision (Alais and Burr 2019). In this way, it has been denoted that when sensory evidence is weak, past experience dominates behavior. Conversely, reliable sensory evidence dominates past experience (Schneider et al. 2020). It could be argued that the central nervous system is challenged with a higher level of uncertainty in instances with an absence of helpful sensory cues (e.g., manipulating a T-shaped object with an off-centered mass) than those with helpful cues (e.g., manipulating an L-shaped object with an off-centered mass). Our results here suggest that such situations with incongruent cues, and higher uncertainty, require the system to rely increasingly on prior knowledge and earlier input from ventral regions, which have previously been evidenced to be important in memory of object properties (Gallivan et al. 2014). Moreover, these results add to the growing body of literature that supports the interaction of ventral and dorsal regions for dexterous object manipulation. We extend these findings by suggesting the level of precursory ventral input depends on whether object properties are compatible or incompatible (and more uncertain) with that afforded by visual shape alone.

Further work is needed to disentangle the contribution of visual cues and sensorimotor memory when both are available to guide dexterous actions. Once experience is acquired with an L-shaped object, our design was not optimized to dissociate whether pattern distances are driven by visual shape (L or reversed L) or CoM (left or right) since they are inextricably linked. It is also possible that pattern distances within a given ROI are reflective of shape at some time points and CoM at others. On the one hand, objects triggering both visual cues and sensorimotor memory might still access a sensorimotor memory of its CoM or torque via congruent visual information alluding to its CoM (perhaps more swiftly). This might be the case in some ROIs more so than others. In support of this supposition, a recently published study suggests that occipitotemporal cortex is sensitive to conceptual rather than shape-varying properties of objects (He et al. 2020). On the other hand, recent behavioral work suggests that both sensorimotor memories and visual cues, when available, contribute to torque generation, but the latter makes a larger contribution (Schneider et al. 2020). Our results show that all regions, including dorsal regions, were sensitive to visual shape differences, as has also previously been shown (Fabbri et al. 2016; Bracci et al. 2017; Freud et al. 2017a, 2017b). Thus, it is conceivable for these cues, when they are available, to be encoded preferentially and to contribute to successful object manipulation, and for there to be less reliance on ventral input when such object properties can be visually inferred online (Gallivan and Goodale 2018; de la Malla et al. 2019).

In summary, this study has given insight into the unique temporal differences in the emergence of CoM-specific activity patterns in ventral, dorsal, and cerebellar regions in planning to dexterously manipulate objects with and without the availability of salient visual cues. An earlier emergence of CoM-specificity, particularly in ventral regions, seems key in object manipulation with added uncertainty as a result of incongruent object features.

## Notes

We thank Mario Mendoza, Danny Toomey, and Naomi Meave Ojeda for their assistance with data collection. *Conflict of interest*: The authors have no competing financial interests.

## Funding

National Health and Medical Research Council (NHMRC CJ Martin Biomedical Fellowship, GNT1110090, MM) and the Rutherford Fett Fund (S.T.G).
